# Ontologies in modelling and analysing of big genetic data

**DOI:** 10.18699/vjgb-24-101

**Published:** 2024-12

**Authors:** N.L. Podkolodnyy, O.A. Podkolodnaya, V.A. Ivanisenko, M.A. Marchenko

**Affiliations:** Institute of Cytology and Genetics of the Siberian Branch of the Russian Academy of Sciences, Novosibirsk, Russia Institute of Computational Mathematics and Mathematical Geophysics of the Siberian Branch of the Russian Academy of Sciences, Novosibirsk, Russia Novosibirsk State University, Novosibirsk, Russia Kurchatov Genomic Center of ICG SB RAS, Novosibirsk, Russia; Institute of Cytology and Genetics of the Siberian Branch of the Russian Academy of Sciences, Novosibirsk, Russia; Institute of Cytology and Genetics of the Siberian Branch of the Russian Academy of Sciences, Novosibirsk, Russia Kurchatov Genomic Center of ICG SB RAS, Novosibirsk, Russia; Institute of Computational Mathematics and Mathematical Geophysics of the Siberian Branch of the Russian Academy of Sciences, Novosibirsk, Russia Novosibirsk State University, Novosibirsk, Russia

**Keywords:** ontologies, big data analysis, bioinformatics, systems biology, deep learning, interpretability, онтологии, биоинформатика, системная биология, анализ больших данных, глубокое обучение, интерпретируемость

## Abstract

To systematize and effectively use the huge volume of experimental data accumulated in the field of bioinformatics and biomedicine, new approaches based on ontologies are needed, including automated methods for semantic integration of heterogeneous experimental data, methods for creating large knowledge bases and self-interpreting methods for analyzing large heterogeneous data based on deep learning. The article briefly presents the features of the subject area (bioinformatics, systems biology, biomedicine), formal definitions of the concept of ontology and knowledge graphs, as well as examples of using ontologies for semantic integration of heterogeneous data and creating large knowledge bases, as well as interpreting the results of deep learning on big data. As an example of a successful project, the Gene Ontology knowledge base is described, which not only includes terminological knowledge and gene ontology annotations (GOA), but also causal influence models (GO-CAM). This makes it useful not only for genomic biology, but also for systems biology, as well as for interpreting large-scale experimental data. An approach to building large ontologies using design patterns is discussed, using the ontology of biological attributes (OBA) as an example. Here, most of the classification is automatically computed based on previously created reference ontologies using automated inference, except for a small number of high-level concepts. One of the main problems of deep learning is the lack of interpretability, since neural networks often function as “black boxes” unable to explain their decisions. This paper describes approaches to creating methods for interpreting deep learning models and presents two examples of self-explanatory ontology-based deep learning models: (1) Deep GONet, which integrates Gene Ontology into a hierarchical neural network architecture, where each neuron represents a biological function. Experiments on cancer diagnostic datasets show that Deep GONet is easily interpretable and has high performance in distinguishing cancerous and non-cancerous samples. (2) ONN4MST, which uses biome ontologies to trace microbial sources of samples whose niches were previously poorly studied or unknown, detecting microbial contaminants. ONN4MST can distinguish samples from ontologically similar biomes, thus offering a quantitative way to characterize the evolution of the human gut microbial community. Both examples demonstrate high performance and interpretability, making them valuable tools for analyzing and interpreting big data in biology.

## Abstract (ru)

Для систематизации и эффективного использования огромного объема экспериментальных данных, накопленных в области биоинформатики и биомедицины, необходимы новые подходы, основанные на онтологиях, включая автоматизированные методы семантической интеграции гетерогенных экспериментальных данных, методы создания больших баз знаний и самоинтерпретируемые методы анализа больших разнородных данных на основе глубокого обучения. В статье кратко представлены особенности предметной области (биоинформатика, системная биология, биомедицина), формальные определения понятия онтологии и графов знаний приведены примеры применения онтологий для семантической интеграции гетерогенных данных и создания больших баз знаний, а также интерпретации результатов глубокого обучения на больших данных. В качестве примера успешного проекта описана база знаний Gene Ontology, которая помимо терминологических знаний и аннотаций генов (GOA) включает модели причинных влияний (GO-CAM). Это делает ее полезной не только для геномной биологии, но и для системной биологии, а также для интерпретации крупномасштабных экспериментальных данных. Обсуждается подход к созданию больших онтологий с использованием шаблонов проектирования на примере онтологии биологических атрибутов (OBA). Здесь большая часть классификации автоматически вычисляется на основе ранее созданных эталонных онтологий с помощью автоматизированного логического вывода, за исключением небольшого числа высокоуровневых понятий. Одной из основных проблем глубокого обучения является отсутствие интерпретируемости, поскольку нейронные сети часто функционируют как «черные ящики», не способные объяснить свои решения. В нашей статье описаны подходы к созданию методов интерпретации моделей глубокого обучения и представлены два примера самообъясняемых моделей глубокого обучения на основе онтологий. Модель Deep GONet, которая интегрирует Gene Ontology в иерархическую архитектуру нейронной сети, где каждый нейрон представляет биологическую функцию. Эксперименты с наборами данных диагностики рака показывают, что Deep GONet легко интерпретируется и обладает высокой производительностью для различения раковых и нераковых образцов. Модель ONN4MST, использующая онтологии биома для отслеживания микробных источников образцов, ниши которых ранее были мало изучены или неизвестны, и обнаружения микробных загрязнителей. ONN4MST может отличать образцы от онтологически близких биомов и, таким образом, предлагает количественный способ охарактеризовать развитие микробного сообщества кишечника человека. Оба примера демонстрируют высокую производительность и интерпретируемость, что делает их ценными инструментами для анализа и интерпретации больших данных в биологии.

## Introduction

The term “Big Data” refers to voluminous datasets that are characterized by significant size, diversity, and complexity, making them difficult to process and analyze using traditional methods. Moreover, such data are often incomplete and uncertain, which complicates the task of controlling their quality and accuracy (Qaiser, Ghulam, 2023).

The emergence of qualitatively new research opportunities based on high-throughput experimental technologies such as massively parallel DNA sequencing, multilocus genotyping, multiparametric gene expression profiling using DNA chips, ChIP-on-chip technology, as well as proteomic and metabolomic technologies, has led to the accumulation of unprecedentedly large volumes of experimental data and knowledge (Stephens et al., 2015). The heterogeneity of molecular biological information and its complexity complicate the analysis, systematization and application of these data to solve specific problems in bioinformatics, biotechnology, pharmacology and personalized medicine.

New approaches to big data processing are required to master, systematize and effectively use huge amounts of data. In particular, this includes automated methods for the semantic integration of heterogeneous data, one of the key stages of which is the harmonization of domain concepts, as well as methods for describing and using them. A coordinated description of a specific domain is called an ontology.

Ontologies allow concepts to be represented in a format suitable for machine processing and act as an intermediary between the user and the information system, as well as between members of the scientific community when exchanging data. Thus, ontologies are becoming an important tool in bioinformatics and systems biology, facilitating the semantic integration of experimental data and knowledge in order to create a “unified picture of the world”. In addition, they help solve problems arising in the analysis of big data, overcoming heterogeneity and deficiencies in data quality, and improving the interpretation of deep learning results. Ontologies increase the scalability and efficiency of processing large amounts of information, which makes them indispensable in modern scientific research.

Earlier, the review (Podkolodnyy et al., 2016) presented examples of ontologies describing biological systems at various levels of organization of living systems. This article will present examples of the application of ontologies for the integration of heterogeneous data and the creation of large knowledge bases, as well as the interpretation of data analysis results.

## Formal representation of ontologies 

In computer science, the term “ontology” refers to a conceptual model that represents objects, their properties, and the relationships between them (Chandrasekaran et al., 1999). An ontology includes a set of concepts (terms) of a particular subject area and their definitions, as well as all the information associated with these concepts, such as properties, relations, constraints, axioms, and assertions. This information is necessary for describing and solving problems in the chosen subject area (Podkolodnyy et al., 2016).

Thus, a formal model of an ontology is represented as an ordered triple of finite sets O = <lt;T, R, F>, where T is a finite and non-empty set of classes and concepts (concepts, terms) of the subject area considered in a certain context (in our case: bioinformatics, systems biology, biotechnology, and biomedicine); R is a finite set of relations between concepts of a given subject area; F is a finite set of interpretation functions defined by concepts and/or relations of the ontology O, as well as axioms used to model statements that are always true. This constrains the interpretation and ensures the correct use of concepts

One of the most effective approaches to describing and using domain knowledge is descriptive logics (DL), which define a formal language for describing concepts (concepts, classes, categories, or entities) and relationships between them (called roles), as well as for formulating statements of facts and queries about them, including satisfiability and inclusion checking. In addition, DL includes constructors (operations) for creating conceptual expressions, such as conjunction, disjunction, and relation definition.

From the point of view of descriptive logic, two main categories of knowledge can be distinguished in the domain knowledge base. The first category includes general knowledge about a set of classes of concepts, their properties, and relationships between them, which is referred to as terminological knowledge, or T-Box. The second category covers knowledge about individual objects (instances of classes), their properties, and relationships with other objects, known as assertional knowledge, or A-Box. Thus, the T-Box describes the subject area at the level of abstract concepts, while the A-Box focuses on specific data, representing a database. It is important to note that both components of the knowledge base are interconnected and complement each other.

Knowledge graphs (KGs) are often used to systematically model complex systems, organisms, and diseases, as well as to represent knowledge in bioinformatics and systems biology. According to the definition presented in (Callahan et al. 2024), a knowledge graph is a data structure that represents multiple heterogeneous entities and different types of relationships between them. This structure serves as an abstract framework capable of generating new knowledge and identifying and resolving discrepancies or contradictions, making it useful for a variety of problems and scenarios.

There are three types of knowledge graphs, depending on the complexity of the representation and the functionality of use:

**Simple graphs** are the most common and basic type of graphs. In such graphs, entities are represented as nodes, and edges are used to model the relationships between them. Simple graphs usually lack formal semantics for edges and nodes, which makes them easy to use, but limits the possibilities for deeper analysis and interpretation of data.

**Hybrid graph**or property graph. Hybrid graphs are designed to model entities and their relationships using a combination of standard network representations and formal semantics, such as Resource Description Framework (RDF: https://www.w3.org/RDF) and RDF Schema (RDFS: https://www.w3.org/TR/rdf11-mt). Unlike simple graphs, hybrid graphs based on these standards facilitate integration with other resources and provide greater opportunity for automated knowledge inference. This makes them a more powerful tool for representing and processing complex information.

**Complex graphs**, such as those in the KaBOB system (Livingston et al., 2015; Podkolodnyy et al., 2016), are often built on top of the Web Ontology Language (OWL). Complex graphs are highly expressive, allowing for efficient knowledge generation through deductive inference (Podkolodnyy et al., 2012). Due to its explicit semantics, OWL offers significant advantages over RDF/RDFS in integrating large amounts of biomedical data, making it particularly useful for complex problems in bioinformatics and systems biology.

**Fig. 1.**
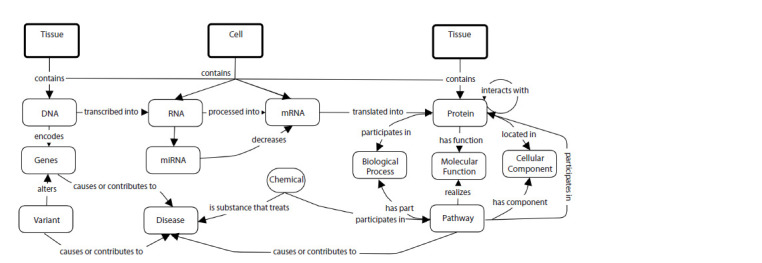
Representation of knowledge about the levels of biological organization underlying the description of human diseases (Callahan, et al., 2024).

As an example, Figure 1 provides a high-level network of the core interrelated biomedical concepts needed to model knowledge about pathways, genetic variants, diseases, and pharmaceutical treatments. At the top level are anatomical entities such as tissues, cells, and biological fluids (compartments) containing genomic entities such as DNA, RNA, mRNA, and proteins. DNA encodes genes, which are transcribed into mRNA and translated into proteins, which have molecular functions, can interact with each other, and participate in pathways and biological processes.

Recently, several software systems have been developed, such as KG-HUB (Caufield et al., 2023), Clinical KG (CKG) (Santos et al., 2022), RTX-KG2 (Wood et al., 2022), BioCypher (Lobentanzer et al., 2023), and Knowledge Base Of Biomedicine (KaBOB) (Livingston et al., 2015; Podkolodnyy et al., 2016), which provide broad functionality for creating and using knowledge graphs in bioinformatics and biomedicine, including the integration of large heterogeneous data.

The work (Callahan et al., 2024) describes the semantic ecosystem PheKnowLator (Phenotype Knowledge Translator) for automating the construction of ontological KGs with a fully customizable knowledge representation. The ecosystem includes various components for creating and using KGs to solve various applied problems, as well as pre-built KGs.

## Integration of big data and creation of knowledge bases based on ontologies

Currently, in the field of bioinformatics, systems biology, agrobiology, biomedicine, more than a thousand ontologies have been developed that can be used to describe and integrate knowledge, analyze data, and infer new knowledge (https://bioportal.bioontology.org/ontologies).

As an example of one of the most successful projects for creating ontologies and, based on this, creating a knowledge base, we can cite the Gene Ontology (GO) project (http://www.geneontology.org/). GO describes current knowledge about the types of functional characteristics (more than 40 thousand concepts in total) that a gene product may have

GO consists of 3 sections:

1. Molecular function – an elementary molecular activity or role that a gene or gene product can play in any biological processes. A total of 10,365 terms are described (https:// geneontology.org/stats.html. Accessed 2024-09-08).

2. Biological process (a total of 26,552 terms are described. Accessed 2024-09-08) – a “biological program” that includes a set of molecular events or activities that act in a coordinated manner to achieve a specific result and relate to the functioning of integrated living units: cells, tissues, organs, and organisms. Unlike a function, a process must have several different stages with a defined beginning and end.

3. Cellular component – a part of the anatomical structure that describes the localization of a gene or its product in an organism, at the levels of cellular structures and macromolecular complexes or groups of gene products. A total of 4,022 terms are described (accessed 2024-09-08).

The main relationships between concepts used in GO include the simple class-subclass relationship (is_a), the part-whole relationship (part_of), the regulates, positively_ regulates, and negatively_regulates relationships that describe relationships between biological processes, molecular functions, or biological properties. The transitivity property of the relationships used in GO allows one to construct a lattice of relationships between concepts and perform logical inference about the properties of concepts and their relationships (Podkolodnyy et al., 2016).

A knowledge base has been created based on GO, which in addition to terminological knowledge (GO gene ontology) includes the results of GOA gene annotation (Gene Ontology Annotation – http://www.ebi.ac.uk/GOA), i. e. knowledge about individual objects – genes and their products (Huntley et al., 2015). Currently, GOA includes more than 7.6 million GO annotations for almost 1.54 million proteins and more than 4.4 thousand species of organisms.

Initially, at the early stage of GO development, annotation of a gene or its product (protein or RNA) was carried out independently by molecular functions, biological processes or cellular components. In order to obtain information about the function of a gene or its product (RNA, protein) in a particular biological process and a particular cellular structure, it was necessary to develop another component of the GO knowledge base – the GO-CAM model of causal influences between gene products (Thomas et al., 2019)

**Fig. 2.**
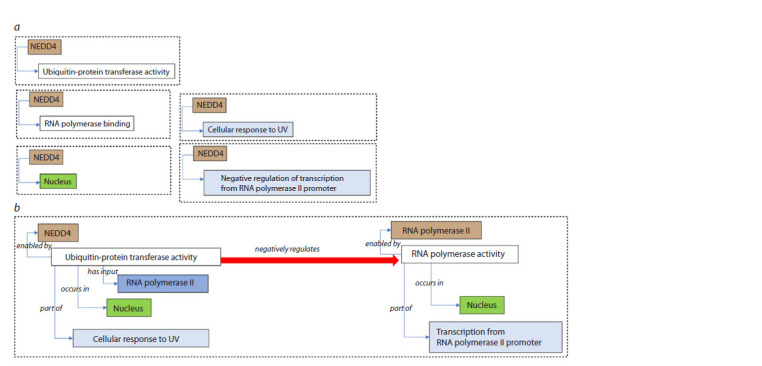
The same biological model of how NEDD4 represses RNA transcription in response to UV-induced DNA damage described in two ways: a – as a set of disparate GO annotations, each capturing a partial description of the overall function; b – as a GO-CAM schema linking the GO annotations into a structured model of NEDD4 function, including the effect of NEDD4 activity on the activity of the RNA polymerase II macromolecular complex (Thomas et al., 2019).

GO-CAM links several GO annotations together to create models of biological processes that connect the activities of more than one gene product together into causal networks and allow specification of the biological context (e. g. cell/ tissue type) in which the activities occur. As an example, the same biological model describing how the E3 ubiquitinprotein ligase NEDD4 represses RNA transcription in response to UV-induced DNA damage can be represented in two ways: as a set of disparate GO annotations, each capturing a partial description of the overall function (Fig. 2a), and as a GO-CAM scheme linking the GO annotations into a structured model of NEDD4 function, including the effect of NEDD4 activity on the activity of the RNA polymerase II macromolecular complex (Fig. 2b) (Thomas et al., 2019).

The basic unit of GO-CAM is the gene product activity unit, which combines the GO MF (molecular activity) annotation, together with the GO CC (cellular component) and GO BP (biological process) annotations, which provide the biological context of the activity. The context can be further specified by other ontologies, including Cell Type Ontology (Diehl et al., 2016), tissue/anatomical location (using several different ontologies depending on the species, e. g. the integrated cross-species anatomy ontology covering animals and merging several species-specific ontologies – Uberon (https://obophenotype.github.io/uberon/) (Mungall et al., 2012), or non-animal ontologies such as Plant ontology (https://planteome.org/) (Cooper, Jaiswal, 2016), or a description of a time period (e. g. biological phase GO). Activity units are related to each other by cause-and-effect relationships from the Relationship Ontology (Smith et al., 2005).

Causal networks in GO-CAM models also enable entirely new applications, such as network analysis of genomic data and logical modeling of biological systems. In addition, the models may also prove useful for pathway visualization. For example, the activity-based GO-CAM representation is compatible with the “activity flow diagrams” of the Systems Biology Graphical Notation (SBGN) standard (Bergmann et al., 2020).

GO-CAM thus provides the opportunity to use the massive GO and GOA knowledge base accumulated over the last 20 years as a basis not only for genomic biology representation of gene function, but also for a broader representation of systems biology and its novel applications to the interpretation of large-scale experimental data

## An example of GO analysis of genes of the associative gene network of rheumatoid arthritis

Earlier, the Institute of Cytology and Genetics SB RAS developed the ANDSystem software and information system for the automated extraction of medical and biological knowledge from scientific publications and a large number of biological and biomedical factual databases (Ivanisenko et al., 2015, 2019). The ANDSystem knowledge base is a unique resource containing formalized information in the form of associative gene networks (knowledge graphs) with almost 44 million interactions of various types between molecular genetic objects.

The original ontology underlying ANDSystem provides a very detailed description of the subject area. The ANDSystem knowledge base describes molecular genetic objects (proteins, genes, metabolites, microRNA), biological processes, phenotypic traits, drugs and their side effects, diseases, etc., as well as more than 25 types of interactions between these objects, including: physical interactions with the formation of molecular complexes (protein/protein, protein/DNA, metabolite/protein); catalytic reactions and proteolytic events involving a substrate/enzyme/product; regulatory interactions, functions/activities, transport and stability of proteins, metabolites and drugs, regulation of protein translation involving miRNA, regulation of biological processes and phenotypic traits involving proteins, metabolites and drugs; associative interactions of genes, proteins, metabolites, biological processes, phenotypic traits with diseases, etc

An example of a typical task using ANDsystem is the reconstruction of an associative gene network (knowledge graph) of rheumatoid arthritis (RA) containing 1,025 genes/ proteins and more than 20 thousand interactions between them. Analysis of the overrepresentation of biological process terms in Gene Ontology for many rheumatoid arthritis genes, performed using the DAVID system (https://david. ncifcrf.gov/tools.jsp) revealed 376 biological processes statistically significantly associated with rheumatoid arthritis (see the Table). The p-values were calculated based on the hypergeometric distribution. The Bonferroni correction was used to account for multiple testing

**Table 1.**
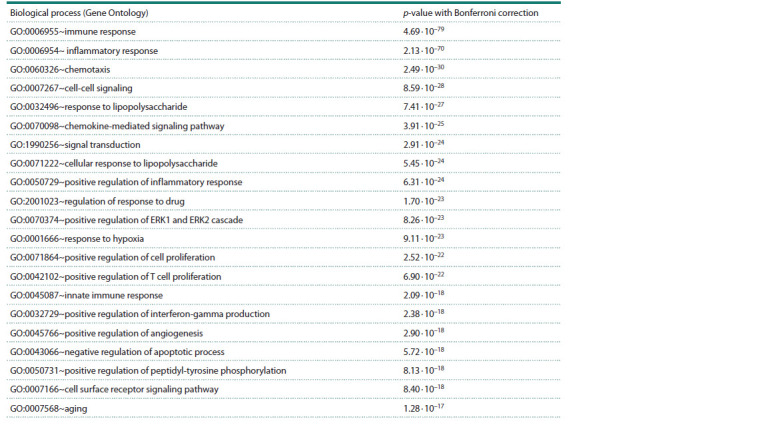
List of the first 21 biological processes statistically most significantly associated with rheumatoid arthritis

**Fig. 3.**
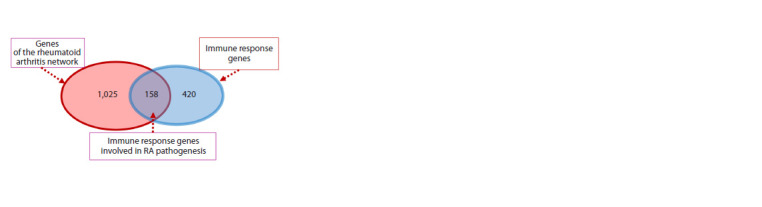
Venn diagram describing the intersection of genes of the rheumatoid arthritis network and immune response genes (associated with the term GO:0006955~immune response).

Let us consider in more detail the GO:0006955~immune response process, which has the lowest p-value, i. e. is most significantly associated with rheumatoid arthritis. Gene Ontology describes 420 genes associated with the “GO:0006955~immune response” term. 158 of them are present in the association network of rheumatoid arthritis (Fig. 3). For random reasons, such a large number of genes can be expected with a very low probability ( p-value with Bonferroni correction < 4.69 · 10–79), which indicates a high significance of the relationship between rheumatoid arthritis and the immune response process and indicates the most important role of the immune system in the pathogenesis of this disease.

The Table presents the list of the first 21 biological processes associated with rheumatoid arthritis and sorted by statistical significance ( p-value with Bonferroni correction). Most of these terms are somehow related to the immune response and inflammation processes, which play an important role in the pathogenesis of rheumatoid arthritis. These processes are not independent.

Thus, the term “GO:0006955~immune response” is associated with such terms from this table as “GO:0045087~innate immune response”, “GO:0032729~positive regulation of interferon- gamma production”, “GO:0060326~chemotaxis”, “GO:0042102~positive regulation of T cell proliferation”, “GO:1990256~signal transduction” and others.

Similarly, the process “GO:0006954~inflammatory response” is associated with the terms “GO:0032496~response to lipopolysaccharide”, “GO:0050729~positive regulation of inflammatory response”, “GO:1990256~ signal transduction”, “GO:0001666~response to hypoxia”, “GO:0045766~positive regulation of angiogenesis”. And even the term “GO:0007568~aging” is related to the term “GO:0006954~inflammatory response”, since one of the mechanisms of aging is chronic non-infectious inflammation.

These results on the example of rheumatoid arthritis indicate that the approach to identifying genes associated with a specific disease using ANDsystem and further GO analysis of this group of genes allows us to identify key biological processes involved in the pathogenesis of this disease

## Using ontology design patterns to integrate phenotype and biological attributes ontologies

Ontologies with logically rich axiomatization provide powerful capabilities such as automated reasoning, classification, and logical queries. However, manually creating such ontologies is extremely expensive and requires annotators to be not only domain experts but also have knowledge of logical modeling (Slater et al., 2020).

A popular approach to solving this problem is to use design patterns and template systems for logical axioms (Osumi-Sutherland et al., 2017). This allows separating the curation of reference terms used for logical definitions from their precise axiomatic picture. The central idea is to use a small number of axiom templates that implement design patterns, which can be created and maintained by logic experts, and for content curators to focus on selecting appropriate filler terms (e. g., terms from the Uberon ontology for defining anatomical attributes).

The Biological Attributes Ontology (OBA) is a standardized framework for observable attributes that are characteristics of organisms or parts of organisms (Stefancsik et al., 2023). Unlike most phenotypic ontologies, in OBA, the logical axioms define general attributes without reference to any specific phenotypic changes or states.

OBA was created using the Entity-Quality (EQ) design pattern, in which a phenotypic quality (Q), such as “height”, “mass”, or “amount” from the Phenotype and Trait Ontology (PATO) (Gkoutos et al., 2005), is combined with an entity (E), such as an anatomical or chemical entity, to form the concept of a “biological attribute” called a “trait”. For example, the concept “blood glucose amount” (OBA:VT0000188) includes the class “amount” (PATO:000070), which defines the glucose characteristic – “glucose” (CHEBI:17234) in the blood – “blood” (UBERON:0000178).

Currently, OBA uses ten feature patterns from the Dead Simple OWL Design Patterns (DOS-DP) (Osumi-Sutherland et al., 2017). They were chosen because they cover most of the anatomical, chemical and cellular attributes that are central to genomics data integration.

A rich logical axiomatization based on design patterns is needed to ensure compatibility with existing phenotype ontologies and other data types, such as anatomical, chemical and biological data on metabolic pathways and gene networks

Most attributes in OBA are inferred using OWL. These inferred definitions use terms from relevant reference ontologies such as Uberon (Mungall et al., 2012) or Chebi (Hastings et al., 2016). Except for a small number of high-level concepts, most of the classification in OBA is automatically computed based on the classifications of various reference ontologies, using automated inference. There are two advantages to this approach: first, no concepts need to be manually classified, which significantly reduces the cost of curating the classification while increasing its completeness. Second, multiple links to reference ontologies can be used for a wide variety of applications, including querying (e. g., retrieving all data where the morphology of a part of the renal system is affected), knowledge graph integration (e. g., automatic linking to phenotypic anomalies from widely used ontologies such as the human phenotype ontology (HPO) or mammalian phenotype ontologies (MP)), and knowledge inference (e. g., inferring missing data) (Dececchi et al., 2015).

## Application of ontologies to interpret deep learning

Deep learning (DL) has clearly demonstrated its effectiveness in solving problems in the field of genomics, proteomics, biomedicine, including analysis and automatic functional annotation of DNA, RNA and protein sequences, search for DNA/RNA targets of regulatory RNAs and proteins, prediction of properties and functions of biomolecules, search for 3D protein structure, reconstruction of structures of biomolecules with given properties, prediction of interactions of biomolecules and identification of potential drug candidates on this basis, image processing and analysis, integration of omics data, analysis of complex, heterogeneous and interconnected biological networks (including proteinprotein interaction networks, gene regulatory networks and metabolic pathways, semantic networks), modeling of biological systems and processes, etc. (Li et al., 2019; Sapoval et al., 2022).

One of the key problems of deep learning in bioinformatics, systems biology and modern biomedicine is the lack of interpretability of neural network models, which often function as “black box” models

Interpretability of machine learning algorithms in bioinformatics and biomedicine is important for three main reasons. First, when analyzing complex systems, when there is no theory and a clear decision-making algorithm, it is necessary to understand why the model predicts a given phenotype. Second, it is important to ensure that the model bases its predictions on a reliable representation of the data and does not focus on irrelevant artifacts. Finally, a model with highly accurate predictions may have revealed interesting patterns that biologists would like to study.

In the formal logical sense, interpretation is the mapping of a formal construct onto the entities and their relationships that it represents. In this sense, one can say that one understands a formal construct if one can relate it to relevant entities and propositions in the real world and reason about the consequences. However, it is important to distinguish the understandability of a model from the understandability of why the model is true or how the model was derived from the data, which raises questions about the validity of the model and the understandability of the learning algorithm.

Two main approaches to interpreting black boxes can be distinguished: a posteriori methods and self-explaining models (Adadi, Berrada, 2018). In the a posteriori method, the black box model is first learned and then an interpretive method is used to explain the predictions. However, explanations often do not match how the deep learning algorithm arrives at a solution. In addition, the explanation procedure is a separate method with its own errors that affect the quality of decisions made. Therefore, such an explanation is not always suitable for biomedicine.

It should be noted that interpretability is a concept specific to a particular domain, so there cannot be a universal definition. Very often, in an interpretable machine learning model, constraints are added to the model form so that it is either useful to someone or obeys structural knowledge of the domain, such as monotonicity (Gupta et al., 2015), causality, structural (generative) constraints, additivity (Lou et al., 2013), or physical constraints that come from knowledge of the subject domain (ontologies)

Currently, several works have been published on building self-explanatory neural networks based on gene expression data using Gene Ontology (GO) knowledge. For example, in the work (Bourgeais et al., 2021), a self-explanatory deep learning model called Deep GONet is proposed, integrating Gene Ontology into a hierarchical neural network architecture. This model is based on a fully connected architecture constrained by Gene Ontology annotations, so that each neuron represents a biological function. Experiments on cancer diagnostic datasets show that Deep GONet is easy to interpret and has high performance in distinguishing cancerous and non-cancerous samples.

Another example of an ontology-based self-explanatory neural network is ONN4MST, a generalization of the Ontology- based Neural Network (ONN) computational model for microbial source tracing (Zha, Ning, 2022). The ONN model uses a novel ontology-based approach that rewards predictions that satisfy the “biome” ontology. In other words, the ONN model can use biome ontology information to model dependencies between biomes and estimate the proportion of different biomes in a community sample.

The knowledge discovery capability of ONN4MST has been demonstrated in various source tracking applications. It enables source tracking of samples, the niches of which were less studied previously or unknown, detection of microbial contaminants, and identification of similar samples from ontologically distant biomes, demonstrating the unique importance of ONN4MST in knowledge discovery from a vast number of microbial community samples from heterogeneous biomes.

ONN4MST can distinguish samples from ontologically similar biomes, thus offering a quantitative way to characterize the evolution of the human gut microbial community. In particular, it is shown that the gut microbiome of centenarians differs from that of normal elderly people and shows a youthful pattern (Zha, Ning, 2022).

## Conclusion

The rapid development of experimental technologies in the field of molecular biology has led to the fact that ontological modeling is becoming a basic method in bioinformatics and systems biology for integrating and analyzing heterogeneous experimental data and using them to build mathematical models of molecular genetic systems and processes. The creation of several hundred basic reference ontologies and their verification allows using these ontologies as sources of knowledge for integrating and building complex domain models and knowledge bases aimed at solving specific problems of biomedicine.

Ontologies are of particular importance for interpreting the results of computer predictions obtained using deep learning methods. In order for scientists to trust deep learning, which is often presented as “black box” models, special interpretation methods based on additional knowledge about the subject area or ontologies should be used. Ontologies, patterns of their construction, integration of big data and creation of knowledge graphs play a key role in increasing the interpretability of deep learning models. These tools not only improve the understanding of the results, but also provide higher quality data analysis. With the rapid growth of information volumes and the complexity of deep learning models, the use of ontologies is becoming a necessary step towards creating more transparent and explainable systems.

It can be expected that the new generation of interpretation systems will be able not only to explain the obtained solutions in a way understandable to humans, indicating the quantitative level of uncertainty, but also to suggest additional steps (e. g., additional experiments, clinical studies, etc.) necessary to clarify or reliably confirm their decisions.

## Conflict of interest

The authors declare no conflict of interest.
